# Exposure to Cadmium and Other Trace Elements Among Individuals with Mild Cognitive Impairment

**DOI:** 10.3390/toxics12120933

**Published:** 2024-12-22

**Authors:** Teresa Urbano, Marco Vinceti, Chiara Carbone, Lauren A. Wise, Marcella Malavolti, Manuela Tondelli, Roberta Bedin, Giulia Vinceti, Alessandro Marti, Annalisa Chiari, Giovanna Zamboni, Bernhard Michalke, Tommaso Filippini

**Affiliations:** 1 Environmental, Genetics, and Nutritional Epidemiology Research Center (CREAGEN), Department of Biomedical, Metabolic and Neural Sciences, University of Modena and Reggio Emilia, 41125 Modena, Italy; teresa.urbano@unimore.it (T.U.); marcella.malavolti@unimore.it (M.M.); tommaso.filippini@unimore.it (T.F.); 2 Department of Epidemiology, Boston University School of Public Health, Boston, MA 02118, USA; lwise@bu.edu; 3 Department of Biomedical, Metabolic and Neural Sciences, University of Modena and Reggio Emilia, 41125 Modena, Italy; chiara.carbone@unimore.it (C.C.); manuela.tondelli@unimore.it (M.T.); roberta.bedin@unimore.it (R.B.); giovanna.zamboni@unimore.it (G.Z.); 4 Neurology Unit, Baggiovara Hospital, 41126 Modena, Italy; gvinceti@gmail.com (G.V.); chiari.annalisa@aou.mo.it (A.C.); 5 Clinical Neuropsychology, Cognitive Disorders and Dyslexia Unit, Neuromotor and Rehabilitation Department, AUSL-IRCCS di Reggio Emilia, 42123 Reggio Emilia, Italy; alessandro.marti@ausl.re.it; 6 Research Unit Analytical BioGeoChemistry, German Research Center for Environmental Health, Helmholtz Center Munich, 85764 Neuherberg, Germany; bernhard.michalke@kabelmail.de; 7 School of Public Health, University of California Berkeley, Berkeley, CA 94704, USA

**Keywords:** amyloid ratio, mild cognitive impairment, neurofilament light chain, Tau proteins, trace elements

## Abstract

Background: A limited number of studies have investigated the role of environmental chemicals in the etiology of mild cognitive impairment (MCI). We performed a cross-sectional study of the association between exposure to selected trace elements and the biomarkers of cognitive decline. Methods: During 2019–2021, we recruited 128 newly diagnosed patients with MCI from two Neurology Clinics in Northern Italy, i.e., Modena and Reggio Emilia. At baseline, we measured serum and cerebrospinal fluid (CSF) concentrations of cadmium, copper, iron, manganese, and zinc using inductively coupled plasma mass spectrometry. With immuno-enzymatic assays, we estimated concentrations of β-amyloid 1-40, β-amyloid 1-42, Total Tau and phosphorylated Tau181 proteins, neurofilament light chain (NfL), and the mini-mental state examination (MMSE) to assess cognitive status. We used spline regression to explore the shape of the association between exposure and each endpoint, adjusted for age at diagnosis, educational attainment, MMSE, and sex. Results: In analyses between the serum and CSF concentrations of trace metals, we found monotonic positive correlations between copper and zinc, while an inverse association was observed for cadmium. Serum cadmium concentrations were inversely associated with amyloid ratio and positively associated with Tau proteins. Serum iron concentrations showed the opposite trend, while copper, manganese, and zinc displayed heterogeneous non-linear associations with amyloid ratio and Tau biomarkers. Regarding CSF exposure biomarkers, only cadmium consistently showed an inverse association with amyloid ratio, while iron was positively associated with Tau. Cadmium concentrations in CSF were not appreciably associated with serum NfL levels, while we observed an inverted U-shaped association with CSF NfL, similar to that observed for copper. In CSF, zinc was the only trace element positively associated with NfL at high concentrations. Conclusions: In this cross-sectional study, high serum cadmium concentrations were associated with selected biomarkers of cognitive impairment. Findings for the other trace elements were difficult to interpret, showing complex and inconsistent associations with the neurodegenerative endpoints examined.

## 1. Introduction

Dementia represents a significant public health challenge, as the population ages globally. The global population of individuals living with dementia is expected to rise to 153 million by 2050 [1]. Emerging evidence suggests that not only genetic and lifestyle factors, but also environmental exposures, including heavy metals and trace elements like cadmium (Cd), copper (Cu), iron (Fe), manganese (Mn), and zinc (Zn), are involved in the pathogenesis of cognitive decline and dementia [2]. While Cd is a non-essential metal with no recognized biologic function, Cu, Fe, Mn, and Zn are essential nutrients that also have potential neurotoxic effects, depending on the amount of exposure, the chemical species, and other factors. Understanding the contribution of these trace elements to neurodegenerative processes is crucial for identifying modifiable risk factors and developing targeted interventions.

Cd, a well-known environmental pollutant, has been associated with neurodegeneration in epidemiologic studies [3,4,5,6], potentially due to its capacity to exacerbate oxidative stress and inflammation in the central nervous system, as shown in laboratory studies [7,8,9]. Cu is a trace element involved in several physiological functions, including mitochondrial energy production, oxidative stress regulation, and neurotransmitter synthesis as a cofactor of enzymes such as superoxide dismutase and cytochrome c oxidase [10], though it could also induce neurodegeneration through its toxic and pro-oxidant properties [11,12]. Zn plays a pivotal role in maintaining synaptic plasticity and supporting neurogenesis. It is concentrated in synaptic vesicles and is involved in modulating neurotransmitter release, neuronal signaling, and enzymatic activity [13]. Both Cu and Zn, while being essential for neuronal function, may be detrimental at high concentrations, contributing to amyloid beta aggregation and Tau phosphorylation, the pathological features of Alzheimer’s disease (AD). The same applies to essential elements like Fe and Mn, which at high levels of exposure may be neurotoxic, affecting neurotransmitter synthesis and promoting oxidative damage [14]. Fe is fundamental to several neurological processes, including neuronal development, myelination, and neurotransmitter synthesis and catabolism. However, it also promotes reactive oxygen species protein oxidation, lipid peroxidation, and nucleic acid modification [15,16]. Mn serves as a cofactor for several key enzymes, including manganese superoxide dismutase, which protects cells from oxidative damage by neutralizing superoxide radicals. Mn is also involved in the synthesis of glutamine and glutamate, neurotransmitters critical for brain function [17], but at higher levels Mn may induce neurotoxicity [18,19]. The impact of different trace elements can vary by the brain region affected and is often dose dependent [20]. Fe, the most abundant trace element in the brain, tends to accumulate in the substantia nigra, striatus, and hippocampus, while Zn accumulates in the cortex, hippocampus, and amygdala. High Cu concentrations have been found in the hippocampus and cortex [21], while chronic Mn mainly affects the basal ganglia [22].

We performed a cross-sectional study among patients with MCI to examine associations between the concentrations of Cd, Cu, Fe, Mn, and Zn in serum and cerebrospinal fluid (CSF) and the biomarkers of cognitive decline, including amyloid Aβ1-42/1-40 ratio (reflecting amyloid plaque deposition), Tau proteins (indicating neurodegeneration), and neurofilament light chain (NfL, reflecting axonal injury) [23].

## 2. Methods

### 2.1. Study Population

We enrolled patients consecutively admitted to the neurology departments of Reggio Emilia ASMN Hospital and Modena University Hospital from 2019 to 2021 with a diagnosis of subjective cognitive decline (SCD) or MCI based on history and neuropsychological assessment [24,25,26]. Detailed inclusion criteria were as follows: clinical diagnosis of MCI or SCD based on history and neuropsychological assessment, including the mini-mental state examination (MMSE), in accordance with the last edition of the *Diagnostic and Statistical Manual of Mental Disorders* (DSM-5) criteria [27]; age at recruitment less than 70 years; onset of reported cognitive impairment before the age of 65; ability to read and write in the Italian language for neuropsychological evaluation; presence of a caregiver available to respond to questionnaires as part of the neuropsychological assessment. Exclusion criteria were as follows: failure to provide consent or withdrawal of consent; diagnosis within the last 2 years of stroke or other cerebrovascular disease; cranial trauma or other focal brain injuries; inflammatory central nervous system disease; other neurodegenerative diseases (Parkinson’s disease, Huntington’s, etc.); major psychiatric disorders; pregnancy. The protocol of this study was approved by the Modena (AOU no. 2158/19) and Reggio Emilia (AUSLRE no. 2019/0009686) Ethics Committees.

**Figure 1:**
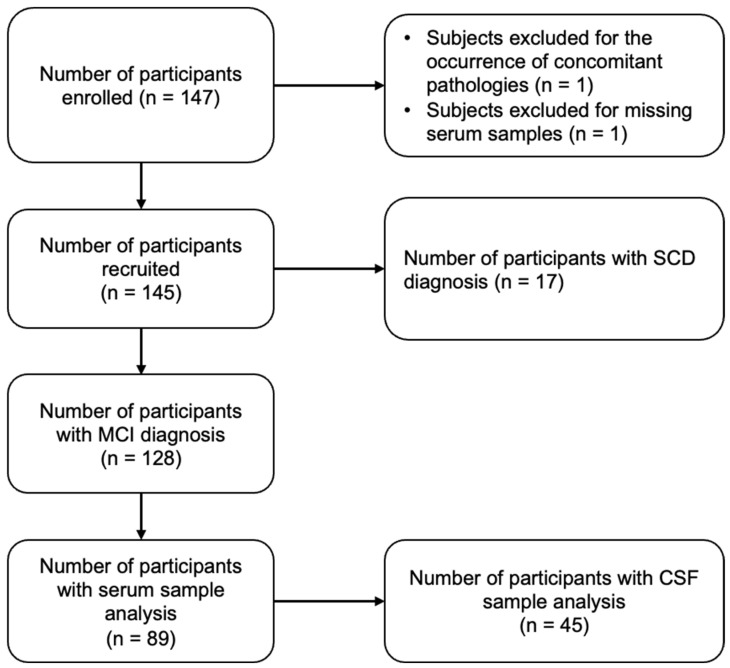
Study flowchart. Abbreviations: CSF, cerebrospinal fluid; SCD, subjective cognitive decline (SCD).

A flowchart of study exclusions is shown in Figure 1. Of the 147 enrolled patients, we excluded two patients with other neurological diseases (e.g., neurosyphilis) and no available serum sample. Of the 145 participants included in the analysis, we eventually assayed trace element concentrations in 103 patients with serum samples. A CSF analysis was performed in only 50 of them due to limited sample availability. Data on each patient’s medical history, sociodemographic information, and lifestyle habits were collected through their medical records and ad hoc project-designed questionnaires. For the purpose of this study, analyses were performed in 89 MCI patients with serum samples and 45 MCI patients with CSF samples.

Patients provided informed consent before undergoing a lumbar puncture to collect CSF samples for diagnostic purposes. Subsequent approval for the use of these samples in research was granted by the Modena Ethical Committee. We collected approximately 6 mL of CSF from each patient under sterile conditions employing an ultraclean technique. Serum samples were also obtained. We promptly stored the samples at −80 °C in polypropylene tubes to ensure preservation. Prior to analysis, we thawed the samples in a refrigerator maintained at 4 °C. Once thawed, they were allowed to reach room temperature, subjected to vortex mixing to ensure homogeneity, and subsequently analyzed.

### 2.2. Biomarkers of Neurodegeneration

The CSF amyloid beta ratio (Aβ1-42/1-40), Total Tau, and phosphorylated Tau 181 (p-Tau181) were assessed with the Lumipulse™ fully automated system using CLEIA (Fujirebio Inc., Ghent, Belgium) following manufacturer instructions. Lumipulse^®^ G β-Amyloid_1-42_, Lumipulse^®^ G β-Amyloid_1-40_, Lumipulse^®^ G Total Tau, and Lumipulse^®^ G p-Tau181 Chemiluminescent Enzyme ImmunoAssays kits (Fujirebio Inc., Ghent, Belgium) were employed. Specific laboratory cut-offs were as follows: amyloid ratio > 0.069 pg/mL, Total Tau < 400 pg/mL, and p-Tau181 < 56.5 pg/mL. Serum and CSF concentrations of NfL were determined using Ella™ microfluidic platform (Bio-Techne, Minneapolis, MN, USA), as previously described [28,29]. Briefly, after 1:2 manual dilution according to the manufacturer’s recommended procedures, samples were loaded into cartridges coated with a capture antibody. Samples were read in triplicate, evaluating intra-assay and inter-assay variability. All analytical procedures were performed by biologists who were blinded to clinical information.

### 2.3. Trace Element (Cd, Cu, Fe, Mn, Zn) Determination

We transferred a 1 mL aliquot of serum and 1 mL of CSF in polypropylene tubes. Samples were transported in a frozen state on dry ice to the laboratory at the Helmoltz Zentrum in Munich where they were maintained in a frozen condition until analysis. We used inductively coupled plasma atomic emission spectrometry (Optima 7300 DV system, Perkin Elmer, Rodgau-Jügesheim, Germany) for Cu, Fe, Mn, and Zn determinations (Schramel 1988), introducing samples with the use of a peristaltic pump connected with a Seaspray nebulizer and a cyclon spray chamber. The measured spectral element lines were Cu: 324.754 nm, Fe: 259.941 nm, Mn: 257.611 nm, and Zn: 213.857 nm. We set RF power to 1400 W, plasma gas at 13 L Ar/min; the nebulizer gas was approximately 0.6 L Ar/min after daily optimization. Since Cd levels were very low, we used inductively coupled plasma sector field mass spectrometry (ICP-sf-MS, “Element 2” from Thermo-Scientific, Bremen, Germany) to analyze 111 Cd [30]. As part of routine quality control, every ten measurements included three blank determinations and a control assessment using a certified standard for all analyzed elements. The final measurements were calculated using a computerized laboratory data management system, which integrated sample measurements with calibration curves, blank determinations, and control standards to ensure accuracy and reliability.

### 2.4. Data Analysis

For data analyses, we used the following routines of Stata statistical software (Stata/MP 18, Stata Corp., College Station, TX, USA, 2024): ‘margins’, ‘mkspline’, ‘regress’, ‘winsor’, ‘xbcrsplinei’, and ‘vioplot’. Of the characteristics reported in Table 1, we created categorical variables for age (<65 and ≥65 years), educational attainment, and body mass index (BMI) (underweight, <18.5 kg/m^2^; normal weight, 18.5–24.9 kg/m^2^; overweight/obese, 25 or more). There were no missing data for covariates, with the exception of BMI (19%) and marital status (12%); the latter covariates were not included in multivariable regression models because they had minimal effects on exposure–outcome associations.

We used crude and multivariable models to assess associations between trace element concentrations in serum and CSF, with the latter analysis being adjusted for age, sex, educational attainment, and MMSE as potential confounders. We analyzed the associations between trace element concentrations and NfL, amyloid ratio, Total Tau, and p-Tau181. We estimated associations using linear fit models with marginal statistics and restricted cubic spline regression models with knots at three fixed percentiles, i.e., 10th, 50th, and 90th of trace element concentrations [31,32].

## 3. Results

Table 1 shows the baseline characteristics of the study population. We originally recruited 145 patients, including 128 MCI. In total, 42% of MCI patients were males, with a median age at enrollment corresponding to 61 years (IQR 56–65). Of the 89 MCI patients with serum trace element data, 45 also had CSF available. No substantial differences in population characteristics were observed between those with (1) only serum and (2) both serum and CSF. We found no appreciable differences in education or MMSE scores. Most patients were less than 65 years old, obtained a high school diploma, were married/partnered, and were overweight or obese.

Table 2 shows serum and CSF trace elements and NfL concentrations along with CSF Total Tau, p-Tau181, and amyloid ratio.

**Figure 2:**
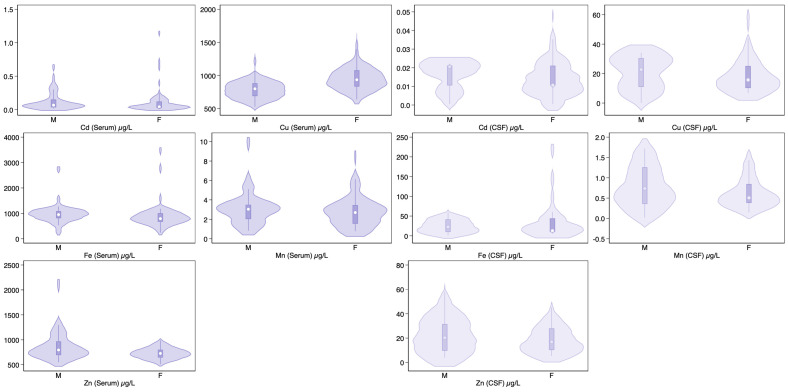
Violin plots distribution of trace element concentrations in serum and cerebrospinal fluid (CSF) according to sex (M, males; F, females). MCI, serum n = 89; CSF n = 45. Abbreviations: Cd, cadmium; Cu, copper; Fe, iron; MCI, mild cognitive impairment; Mn, manganese; NfL, neurofilament light chain; SCD, subjective cognitive decline; Zn, zinc.

The highest concentrations in serum and CSF occurred in the following order: Fe > Cu > Zn > Mn > Cd. Limited differences were instead observed for serum and CSF levels of the trace elements investigated according to sex (Figure 2).

**Figure 3:**
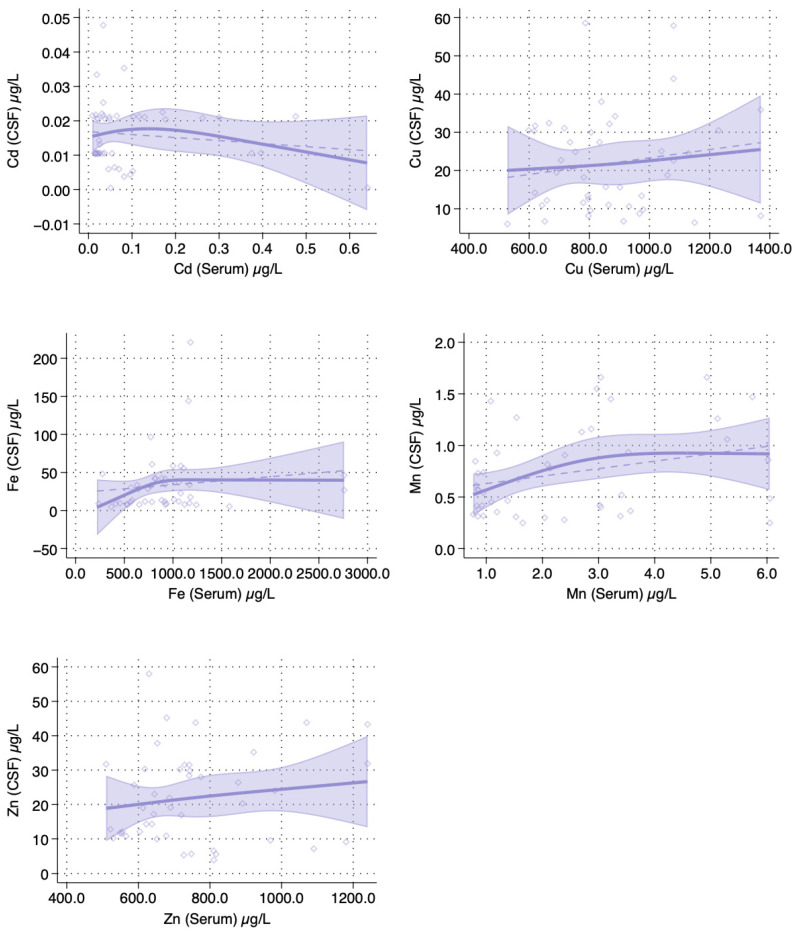
Spline regression analysis of the association between trace element concentration in serum and cerebrospinal fluid among patients with mild cognitive impairment. The solid line indicates the multivariable analysis; the shaded area represents upper and lower confidence interval limits. The dashed line represents association assuming linearity. Diamonds represent individual observations (n = 45). Abbreviations: Cd, cadmium; CSF, cerebrospinal fluid; Cu, copper; Fe, iron; Mn, manganese; NfL, neurofilament light chain; Zn, zinc.

Among 45 patients with data on serum and CSF, we compared serum and CSF concentrations for each of the trace elements. In spline regression analyses among MCI patients, we observed a positive linear association between serum and CSF concentrations of Cu and Zn, and an inverse association for Cd. There was a positive association between Fe concentrations in serum and CSF up to a threshold of 1000 μg/L, beyond which the relation plateaued. For Mn, there was a slight inverted U-shaped association, with a positive monotonic relation between the serum and CSF matrices until 3.0 μg/L and a slight inverse association above that value (Figure 3).

**Figure 4:**
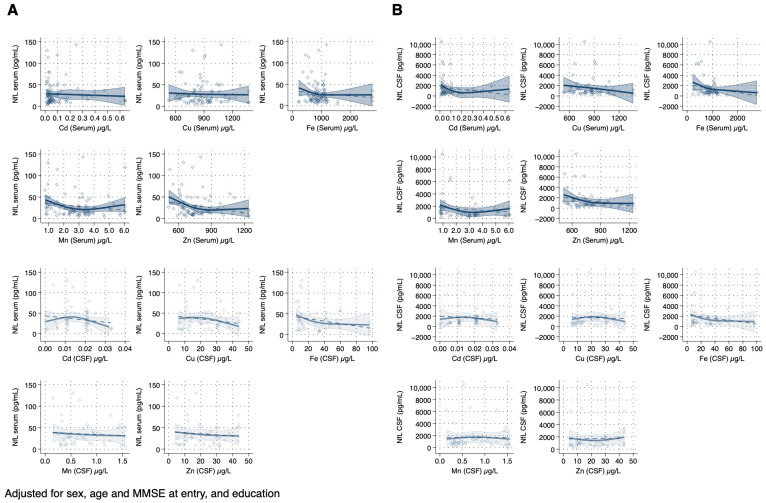
Spline regression analysis of the association between trace element concentration in serum (dark blue) and cerebrospinal fluid (CSF-light blue) with serum (**A**) and CSF neurofilament light (NfL) concentrations (**B**) among patients with mild cognitive impairment. The solid line indicates the multivariable analysis; the shaded area represents the upper and lower confidence interval limits. The dashed line represents the association assuming linearity. Diamonds represent individual observations (serum n = 89; CSF n = 45). Abbreviations: Cd, cadmium; Cu, copper; Fe, iron; MMSE, mini-mental state examination; Mn, manganese; Zn, zinc.

When we examined the association between the trace element concentrations and each study endpoint, in serum, we found little association of Cd and Cu concentrations with NfL, whereas we observed non-linear associations of Fe, Mn, and Zn with NfL. For Fe, we observed an inverse association until 1000 μg/L after which the effect plateaued. For Mn and Zn, we found a U-shaped relation, with an inflection point at 3.0 μg/L and 800 μg/L, respectively (Figure 4A). In CSF, Cd and Cu showed an inverse U-shaped association with NfL concentrations, while Fe showed a monotonic inverse association. For Mn and Zn, the association was almost flat at the lowest exposure levels, while there was an inverse association for Mn above 1.0 μg/L and a positive association for Zn above 20 μg/L (Figure 4B). When comparing associations between trace elements and NfL concentrations across matrices (serum vs. CSF), the associations were substantially similar.

**Figure 5:**
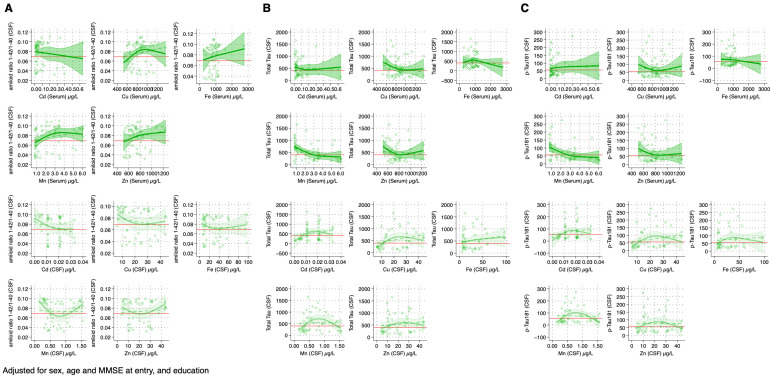
Spline regression analysis of the association between trace element concentration in serum (dark green) and cerebrospinal fluid (CSF—light green) with CSF concentration of amyloid ratio (**A**), Total Tau (**B**) and phosphorylated Tau (p-Tau181) protein (**C**) among patients with mild cognitive impairment. The solid line indicates the multivariable analysis; the shaded area represents the upper and lower confidence interval limits. The dashed line represents the association assuming linearity. Diamonds represent individual observations (serum n = 89; CSF n = 45). Red lines represent laboratory cut-offs (>0.069 for amyloid ratio; <400 pg/mL for Total Tau; <56.5 pg/mL for p-Tau181). Abbreviations: Cd, cadmium; Cu, copper; Fe, iron; MMSE, mini-mental state examination; Mn, manganese; Zn, zinc.

For Cd, we found an inverse association with amyloid ratio, and a positive association with total Tau and p-Tau181. On the contrary, an opposite pattern emerged for Fe, since its serum concentrations were positively associated with amyloid ratio and inversely associated with Tau proteins. Some discrepancies were observed in the association between Cu and the neurodegeneration biomarkers. In fact, Cu was positively associated with amyloid ratio until 800 μg/L after which an inverse relation emerged. Similarly, there was a U-shaped association with Total Tau and p-Tau181, showing an inverse and then a positive association at the same inflection point. Mn concentrations showed an inverse U-shaped association with amyloid ratio, while being inversely, although not linearly, associated with Tau proteins. Zn concentrations were positively associated with amyloid ratio, while demonstrating a U-shaped association with total Tau and an inverse association with p-Tau181, but only until 800 μg/L. Above this value, the association with amyloid ratio flattened, and the association with Total and p-Tau181 became positive. In CSF, Cd concentrations were inversely associated with amyloid ratio, and there was a slight inverse U-shaped association with Tau protein biomarkers. Cu, Mn, and Zn also showed a U-shaped association with amyloid ratio, while showing an inverse U-shaped association with Tau proteins, with inflection points at 20, 0.75, and 25 μg/L, respectively. Fe concentrations were positively and monotonically associated with Total Tau, showing an inverse U-shaped association with p-Tau181, and showing no association with amyloid ratio (Figure 5). Finally, according to the laboratory cut-off values of the neurodegeneration biomarkers used for AD, Cd in serum was the only element found to be inversely associated with amyloid ratio and positively associated with Tau proteins, while higher concentrations of Zn and Cu in serum were positively associated with Total Tau and p-Tau181, respectively. When considering CSF trace element concentrations, none of the elements demonstrated a clear association with amyloid ratio. When considering Total Tau, we found a positive association above 400 pg/mL for all elements, with a linear association for the full range of exposure only for Fe. We observed similar associations for p-Tau181, although the slopes of the spline curves were considerably less steep.

## 4. Discussion

In this cross-sectional study among patients with MCI, some trace elements measured in serum and CSF were positively associated with the biomarkers of neurodegeneration. Most associations were non-linear. Cd in particular was positively associated with neurodegeneration biomarkers, with the exception of NfL, consistent with an ongoing neurodegenerative process. Other trace elements displayed inconsistent and heterogeneous non-linear relations with amyloid and Tau biomarkers, while Mn was the only trace element shown to have a U-shaped association with NfL.

As for the associations between serum and CSF concentrations of trace metals, we found monotonic positive correlations only for Cu and Zn. The inverse or non-linear associations between the concentrations of some elements by matrix (serum vs. CSF) can be attributed to several factors, including altered blood-CSF barrier permeability and the dynamics of the transport of metals across the blood–brain barrier [33,34,35]. For instance, in the case of Fe, homeostatic mechanisms may occur when serum metal concentrations increase without a corresponding rise in CSF. Therefore, when serum metal concentrations rise, the blood–brain barrier may become more restrictive, preventing excess metals from entering the CSF. In addition, neurons and glial cells can actively regulate metal homeostasis through specific transporters and channels, thereby preventing accumulation in CSF [36]. Conversely, the inverse association we observed for Cd between serum and CSF concentrations was unexpected and intriguing, since most of the epidemiological studies conducted to date used urinary levels and biomarkers of exposure [37,38]. Evidence about whether the CSF content may reflect the blood concentrations of this trace element is scarce and some epidemiological studies have raised concerns about the reliability of CSF as a biomarker of exposure [39]. In a previous study, a positive and relatively strong correlation was found between the serum and CSF concentrations of Cd in children, hypothesizing that this trace element exhibits the capacity to access the brain via the nasal mucosa or olfactory pathways, which are not yet fully developed in young children [33]. Our results may differ from those in previous studies due to the different physiological mechanisms in populations of different ages. Cd’s affinity for sulfhydryl groups in serum proteins could lead to a scenario where higher serum concentrations do not correspond to increased concentrations in CSF, hypothesizing a protective role of serum against Cd toxicity [7].

Recent findings have highlighted a potential role of some trace elements such as Cd, a heavy metal potentially linked to an excess risk of many diseases [40] including cardiovascular disorders [41,42,43] and neurodevelopmental disorders [44], in the etiology of different forms of dementia, particularly that are induced by AD [45,46]. Conversely, little evidence is available for MCI, long recognized as a precursor of dementia [47]. Therefore, understanding the environmental and biological factors contributing to MCI progression is essential for developing preventive strategies for dementia [48]. The studies of Cd’s neurotoxic effects have indicated that even low-level exposure can lead to impairments in cognitive performance among older adults [49,50,51]. Several studies have also indicated that Cd has the ability to induce oxidative stress and neuroinflammation, which can disrupt neuronal function and lead to cognitive decline [52,53]. Cd accumulation might also exacerbate some pathological features of AD, such as a reduction in β-amyloid degrading enzymes leading to increased β-amyloid accumulation [54]. In our study, among the trace elements measured in serum, Cd was the only element associated with adverse effects on neurodegeneration biomarkers, as highlighted by the associations found upon exceeding the standard laboratory values of amyloid ratio and Tau proteins. This association emerged at exposure concentrations believed not to be unusually high, given that the median serum Cd concentrations in our study were lower than those measured in the National Health Nutrition Examination Survey [55] or in the Beaver Dam Offspring Study cohort, which found comparable associations between serum Cd and amyloid ratio or Tau proteins [56]. Hence, our results appear to be even more relevant if considering that we observed adverse associations at lower concentrations than previous reports. However, the results for CSF concentrations did not reflect the same associations found for serum Cd, a discrepancy that warrants further investigation.

Fe is the most abundant transition metal and the most investigated in relation to AD. Through Fenton chemistry, Fe can catalyze the formation of reactive oxygen species (ROS), leading to detrimental processes for neuronal integrity, such as lipid peroxidation and protein oxidation [57]. For Fe, we observed an inverse relation between serum and CSF concentrations and neurodegeneration biomarkers, with the exception of a positive association between Fe and total Tau in CSF. The latter result is consistent with previous results highlighting a positive relation between brain Fe deposition and atrophy, cognitive decline, and Tau aggregation in elderly individuals [58,59,60]. A recent systematic review highlighted that lower Fe in blood and greater ferritin in CSF were found among AD patients compared with healthy subjects [61]. In this study, we did not perform Fe speciation analysis, thus we could not ascribe distinct associations to different Fe compounds.

Zn and Cu are nutritionally essential trace elements that play significant roles in brain function, but they may also have neurotoxic properties at high concentrations. Particularly in the case of Cu, it operates as a catalyst of redox reactions and, in the context of dementia and AD, it has been shown to interact with β-amyloid peptides, promoting oxidative damage to neuronal membranes and facilitating their aggregation [62,63]. Moreover, Cu is known to promote Tau protein hyperphosphorylation and it has been linked to the activation of inflammatory pathways, further compounding oxidative stress and neuronal injury [11,64]. While being essential for synaptic plasticity and cognitive function at low concentrations and capable of β-amyloid-induced toxicity suppression, at high levels Zn binds β-amyloid, thereby enhancing fibrillary aggregation and leading to neurodegeneration [65,66]. We found evidence of some detrimental effects of these two trace metals in relation only to Tau proteins at the highest concentrations in serum, consistent with a previous case–control study finding a positive correlation of non-ceruloplasmin serum Cu with CSF total Tau levels [67]. A previous meta-analysis indicated that serum concentrations of Zn were lower among AD patients compared with healthy subjects, even though there was high heterogeneity between studies [68].

With regard to Mn, while necessary for several enzymatic functions, it can be neurotoxic at elevated levels. Recent reviews suggested that Mn exposure may disrupt iron homeostasis, and its accumulation contributes to oxidative stress and mitochondrial dysfunction [69,70]. The competition between Mn and other trace metals for transport mechanisms in the brain can exacerbate these effects, leading to the further dysregulation of metal homeostasis and neurotoxicity [71]. In a previous study, Mn was slightly positively associated with the risk of cognitive dysfunction among people over 60 years old [72]. However, a 2017 meta-analysis reported a decrease in serum Mn concentrations among individuals with cognitive impairment including AD and MCI subjects compared with healthy controls [73]. In our study, Mn was not associated with neurodegeneration, being inversely and positively associated with Tau proteins and amyloid ratio, respectively.

While some studies in the literature reported the association between trace metals and classical biomarkers of AD pathology used in clinical practice, few studies investigated their associations with NfLs. NfLs are among the most promising biomarkers for detecting neuroaxonal damage across a broad spectrum of neurological disorders. Its concentrations in CSF and blood serve as reliable indicators of neuronal injury and degeneration, reflecting the extent of underlying neural pathology [28,74]. Elevated NfL concentrations have been consistently associated with various forms of dementia, including AD and frontotemporal dementia [75]. In a recent study, Cd in serum was found to be positively associated with NfL concentrations [56], similar to what was observed in our study, where Cd in serum was slightly positively associated only with NfL in CSF at the highest levels. No associations were reported for Cd in CSF. Cu, Fe, Mn, and Zn were positively associated with NfL in CSF in the only study conducted to date [76]. However, we found evidence of inverse or null associations for Cu, Fe, and Zn with NfL. Mn in serum was the only trace metal found to be associated through a U-shaped pattern with NfL in both serum and CSF, suggesting that, while essential, it can exhibit neurotoxic effects when present in non-optimal concentrations.

To our knowledge, this study represents the first ever conducted among cognitively impaired subjects with deficits occurring at relatively young ages (<65 years), and one of the few to investigate the association between trace metals and neurodegeneration biomarkers, including NfL. In addition, the use of non-linear dose analysis allowed a comprehensive assessment of the patterns of association. Finally, a CSF analysis of trace metals provides unique insights into the pathophysiological processes that occur in the central nervous system allowing the direct assessment of the brain’s biochemical environment. Some study limitations must be outlined. First, given the study’s cross-sectional design, associations may not be causal. Ideally, we would have collected longitudinal data among disease-free individuals on the progression to dementia to determine temporality. Misclassification would be minimal if the baseline concentrations of metals reflected the patient’s habitual exposure during the years prior to disease onset. We are also currently collecting longitudinal data on the progression to dementia of the individuals of this cohort to better assess temporal relations. Secondly, metal concentrations measured at a single time point may have contributed to the misclassification of exposure, especially if the relevant time window for exposure was years earlier. The half-lives of the metals examined in this study range from some hours to several years, depending on the sample matrix; thus, we made the strong assumption that the concentrations measured at baseline in our study population were reflective of what patients were exposed to before the onset of MCI. In addition, whole blood might have provided a better reflection of long-term metal status in the body compared with serum, which can be influenced by recent dietary intake. Third, few data are available on pharmacokinetic properties of each metal in CSF; therefore, the stability of some metals in this matrix is uncertain. Fourth, our study population was relatively small, which reduced the precision of our effect estimates and could affect the generalizability of our findings. Finally, we could not rule out potential unmeasured or residual confounding, which may have affected the validity of the observed associations.

## Figures and Tables

**Table 1:** Characteristics of the study population (only mild cognitive impairment (MCI) participants) according to availability of cerebrospinal fluid (CSF) samples.

	MCI Participants (n = 128)	MCI with Serum TE Analysis (n = 89)	MCI with CSF TE Analysis (n = 45)
	Median (IQR)	Median (IQR)	Median (IQR)
Sex (n, %)			
Males	53 (41.4)	36 (40.5)	20 (44.4)
Females	75 (58.6)	53 (59.5)	25 (55.6)
Age at diagnosis (years)	61 (56–65)	61 (56–65)	61 (56–64)
Age in categories (n, %)			
<65 years	90 (70.3)	60 (67.4)	35 (77.8)
≥65 years	38 (29.7)	29 (32.6)	10 (22.2)
Education (years)	11 (8–13)	11 (8–13)	12 (8–13)
Education in categories (n, %)			
Elementary school	11 (8.6)	10 (11.2)	5 (11.1)
Middle school	46 (35.9)	33 (37.1)	12 (26.7)
High school	48 (37.5)	30 (33.7)	18 (40.0)
College or more	23 (18.0)	16 (18.0)	10 (22.2)
Marital status (n, %)			
Single	9 (7.0)	4 (4.5)	3 (6.7)
Married/unmarried	83 (64.8)	61 (68.5)	32 (71.1)
Separated/divorced	13 (10.2)	9 (10.1)	2 (4.4)
Widowed	8 (6.3)	6 (6.7)	4 (8.9)
Missing	15 (11.7)	9 (10.1)	4 (8.9)
BMI in categories (n, %)			
<18.5 kg/m^2^	4 (3.1)	3 (3.4)	2 (4.4)
18.5–24.9 kg/m^2^	42 (32.8)	24 (27.0)	11 (24.4)
≥25.0 kg/m^2^	58 (45.3)	46 (51.7)	25 (55.6)
Missing	24 (18.6)	16 (18.0)	7 (15.6)
MMSE score	27 (25–29)	27 (25–29)	27 (25–29)

Abbreviations: BMI, body mass index; LP, lumbar puncture; MMSE, mini-mental state examination; NfL, neurofilament light chain protein; p-Tau181, Tau protein phosphorylated at threonine 181; TE, trace element.

**Table 2:** Distribution of neurofilament light chain (NfL) and trace elements in serum and cerebrospinal fluid (CSF) among patients with mild cognitive impairment (MCI). Values are median and interquartile ranges (IQR).

	CSF (n = 45) Median (IQR)	Serum (n = 89) Median (IQR)
NfL (pg/mL)	934.0 (665.0–1653.0)	19.5 (12.7–35.0)
Amyloid ratio	0.088 (0.050–0.094)	-
Total Tau (pg/mL)	442.0 (215.0–774.0)	-
p-Tau181 (pg/mL)	46.1 (25.8–116.6)	-
Cadmium (µg/L)	0.01 (0.01–0.02)	0.05 (0.03–0.12)
Copper (µg/L)	19.50 (11.00–30.50)	869 (766–974)
Iron (µg/L)	28.50 (9.82–42.50)	864 (694–1070)
Manganese (µg/L)	0.57 (0.38–0.94)	2.74 (1.55–3.50)
Zinc (µg/L)	19.10 (10.90–30.30)	741 (650–856)

## Data Availability

The data presented in this study are available on request from the corresponding author (the data are not publicly available due to privacy restrictions).
